# Mucus plugs in the airways of asthmatic subjects and smoking status

**DOI:** 10.1186/s12931-024-02665-w

**Published:** 2024-01-23

**Authors:** Camille Audousset, Sana Swaleh, Ron Olivenstein, Motahareh Vameghestahbanati, Miranda Kirby, Alexandre Semionov, Benjamin M. Smith, James G. Martin

**Affiliations:** 1 Meakins-Christie Laboratories, Research Institute of the McGill University Health Centre, Department of Medicine, McGill University, Montréal, QC Canada; 2 Department of Physics, Toronto Metropolitan University, Toronto, Canada; 3 Institute for Biomedical Engineering, Science and Technology (iBEST), St. Michael’s Hospital, Unity Health Toronto, Toronto, Canada; 4 Department of Radiology, McGill University Health Centre, Montréal, Canada

## Abstract

**Background:**

Mucus plugs have been described in the airways of asthmatic subjects, particularly those with associated with type 2 inflammation and sputum eosinophilia. In the current study we addressed the question of whether smoking, neutrophilic inflammation and airway dimensions affected the prevalence of mucus plugs.

**Methods:**

In a cohort of moderate to severe asthmatics (*n* = 50), including a group of ex-smokers and current smokers, the prevalence of mucus plugs was quantified using a semi-quantitative score based on thoracic computerized tomography. The relationships between mucus score, sputum inflammatory profile and airway architecture were tested according to patient’s smoking status.

**Results:**

Among the asthmatics (37% former or active smokers), 74% had at least one mucus plug. The median score was 3 and was unrelated to smoking status. A significant but weak correlation was found between mucus score, FEV_1_ and FEV_1_/FVC. Mucus score was significantly correlated with sputum eosinophils. Among former and active smokers, mucus score was correlated with sputum neutrophils. Mucus score was positively associated with FeNO in non-smoking subjects. The lumen dimensions of the main and lobar bronchi were significantly inversely correlated with mucus score.

**Conclusion:**

Airway mucus plugs could define an asthma phenotype with altered airway architecture and can occur in asthmatic subjects with either neutrophilic or eosinophilic sputum according to their smoking status.

**Supplementary Information:**

The online version contains supplementary material available at 10.1186/s12931-024-02665-w.

## Introduction

Asthma is a frequent and heterogeneous pathology comprising different clinical presentations, inflammatory pathways and endotypes [1]. Recently, the presence of mucus plugs in the airways has been proposed as a phenotypic marker of chronic inflammatory airway diseases such as COPD [2, 3] and asthma [4–6]. The presence of chronic mucus plugs in asthmatics airways, quantified by visual scoring of CT scan images, has been associated with increased airway obstruction, higher type 2 (T2) inflammation, and more exacerbations [4–6]. These plugs persist over time and the evolution of their number is correlated with the evolution of respiratory function [5]. Mucus plugs are also associated with inhomogeneous ventilation distribution [7]. In asthmatics with T2 inflammation, there is considerable evidence to suggest a link between eosinophilic inflammation in sputum and the presence of mucus plugs, but the mechanism underlying the persistence of mucus plugs within the same lung segment has not been established. This feature could be explained by increased local T2 inflammation [4].

Local heightened T2 inflammation may be linked to the presence of Charcot-Leyden protein crystals embedded in mucus plugs. Charcot Leyden crystals have been described in the sputum of asthmatics for more than a century and are attributable to crystallization of the eosinophil protein Galectin-10 (Gal-10) [8], formed during a specific death pathway of eosinophils, namely *EETosis* [9]. Additionally, the crystals trigger innate and adaptive immune responses within the airways, serving as an adjuvant promoting T2 immunity [8]. In vitro, the crystals can be dissolved by specific antibodies opening up a potential new therapeutic option for T2 asthma [8]. The involvement of Charcot-Leyden crystals in chronic airway mucus plugging has not yet been studied.

Many asthmatics have neutrophilic sputum but few eosinophils [10]. Whether mucus plugs are common in these asthmatics is less well studied. Beyond T2 inflammation, the presence of mucus plugs in the airways of asthmatics and COPD subjects has been associated with local thickening of the walls of the obstructed bronchi [3]. Interestingly, in COPD, the presence of mucus plugs was correlated with the percentage of neutrophils in sputum and not with the eosinophils, suggesting the possibility of non-T2 factors associated with airway mucus plugging [2].

The objective of this study was to evaluate the association of sputum plug formation with sputum granulocyte characteristics (neutrophilic or eosinophilic), Gal-10 concentrations and the airways architecture in a cohort of well-characterized asthmatic subjects. We included a group of currently smoking asthmatics since the nature of the inflammatory response is more likely to be neutrophilic in these individuals.

## Materials and methods

### Subjects

Subjects were recruited at the Montreal Chest Institute (McGill University Health Centre, Montreal, Qc, Canada). All asthma subjects were diagnosed with moderate to severe asthma according to American Thoracic Society guidelines [11]. Subjects were recruited from two distinct clinical cohorts, a retrospective cohort including difficult-to-treat asthmatic subjects that have been the subject of previous reports [12, 13] and a prospective cohort of smokers and ex-smokers. The inclusion criteria common to the two cohorts were to have an asthma diagnosis made by a pulmonologist and reversibility of the forced expiratory volume in one second (FEV_1_) as defined by an increase of 12% and 200mL post bronchodilator administration or longitudinal variability of FEV_1_. Detailed inclusion criteria for each study are reported in the Supplementary Data. Smoking severe asthmatics were recruited according to the same criteria and were defined as currently using at least 10 cigarettes per day and at least 10 pack-years lifetime smoking history and the onset of asthma was diagnosed by a physician before the age of 40 years. The exclusion criteria were any other known pulmonary disease including a prior diagnosis of COPD, or a respiratory tract infection or exacerbation of asthma within 6 weeks of enrolment in the study. Healthy controls were non-smokers without a physician diagnosis of asthma.

The study was approved by the Research Ethics Board of the McGill University Health Centre. All the subjects included in the previous studies were eligible for the current study with the additional main inclusion criterion, the availability of a chest computerized tomographic scan (CT) with a minimal CT slice thickness of 2.5 mm. The maximal interval between the CT scan and pulmonary function or sample collection was 3 months.

### Quantification of the mucus plugs on the CT scan

The quantification of mucus plugs was performed using a visual scoring system as proposed by Dunican et al. [4]. All 20 bronchial segments were visually inspected. The presence of one mucus plug in a segment counted as one point and the absence for 0. The total lung score was generated by the addition of the score of each lung segment ranging from 0 to 20. A mucus plug was defined as a complete obstruction of the airway. It appears as a tubular density in a parallel plane and as a rounded opacity in the perpendicular plane. A 2 cm peripheral zone of lung from the costal to the diaphragmatic pleura was excluded as previously described [4]. All CT scans were scored independently by a pulmonologist and a chest radiologist from the McGill University Health Centre. First, a set of 10 scans was analyzed and quantified. The results were compared and discussed between the two evaluators to harmonize approaches to analysis and subsequently all the scans (*n* = 51 asthmatics and 4 controls) were evaluated by each of the evaluators, independently. The comparison of the initial scoring exercise resulted in a Kendall correlation τ of 0.66 (*p* < 0.0001). All CT scans with a score difference of > 4 were discussed (*n* = 5) and a consensus was reached for the final scoring. The comparison of the final scores achieved a Kendall correlation τ of 0.84 (*p* < 0.0001).

### Quantification of the airway dimensions

Airway lumen area, bronchial wall thickness, and percentage of wall thickness to outer airway area were assessed on CT scan using commercial software (VIDA Diagnostics Inc., Coralville, IA, USA) by an analyst unaware of other participant information. All airway segments were independently verified by second analyst. The airways were identified and measured from the trachea to the segmental bronchi according to a previously described method [14].

### Sputum induction

Sputum induction was performed using aerosolized hypertonic saline (3.4% and 5%) after the inhalation of salbutamol (200 µg). Sputum processing was performed as previously described [15]. After collection, the expectorate was treated with dithiothreitol (DTT) (Sputalysin 10%, Calbiochem Corp., San Diego, CA) to facilitate dissolution of mucus. A total cell count was performed by haemacytometer. A differential cell count was performed on Diff-Quik stained slides prepared by cytocentrifuge on a total of 400 cells. The sputum supernatant was stored in Eppendorf tubes at − 80^0^C until measurements were performed.

### Sputum measurements

Gal-10 protein was quantified in the sputum supernatant by ELISA (Gal-1010 Kit, Invitrogen Ref# EH204RB) according to the manufacturer’s recommendations.

### Lung function and FeNO assessment

Spirometry and the fraction of exhaled nitric oxide (FeNO) were assessed according to ATS recommendations [16, 17].

### Statistical analysis

The data normality was assessed by the Shapiro-Wilks normality test. For the non-normal data, non-parametric tests were applied and the results were expressed as median [Interquartile range]. For normally distributed data, the results were expressed as a mean +/- standard deviation (SD) and parametric tests were applied. Correlation between variables was evaluated using Spearman’s correlation. Box-and-whisker plots show the median, 25% and 75% quartiles. All data were presented. Agreement between raters was estimated using the *Kendall* rank correlation coefficient. Statistical analyses were performed out with Graph Pad Prism Version 8.4.3 and R (Core Team (2020). R: A language and environment for statistical computing). R Foundation for Statistical Computing, Vienna, Austria. URL https://www.R-project.org/ with Wickham et al., (2019). Welcome to the tidyverse. Journal of Open Source Software, 4(43), 1686, 10.21105/joss.01686.

## Results

### Subject characteristics

**Fig. 1:**
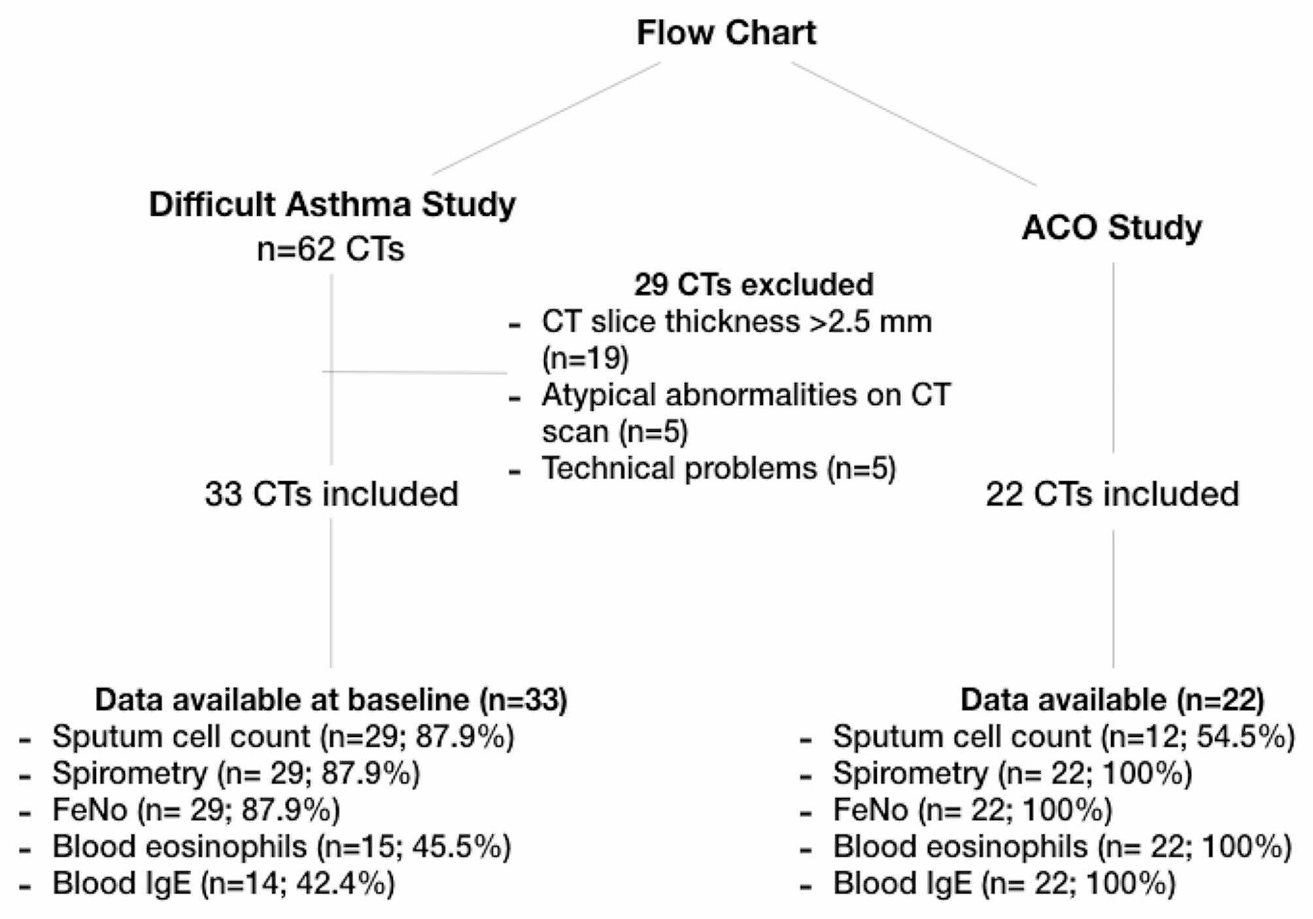
Consort diagram of CT included in the study. Flow chart shows the number of CT scans that were screened and finally included in the final analyses

All subjects in the study of smoking asthmatics (18 asthmatics and 4 healthy subjects) and 33 subjects from the difficult-to-treat asthma study were included. The flowchart of inclusions is summarized in Fig. 1. Demographic and clinical characteristics of the cohort, including 50 asthmatics and 5 healthy subjects, are summarised in Table 1 and in supplemental Table 1. The group with a high mucus score was significantly older compared to other classes of mucus score (*p* = 0,02). 94% (*n* = 39/41) of asthmatics had received treatment with high-dose inhaled corticosteroid therapy and 7 subjects were under treatment with monoclonal antibodies, including omalizumab (*n* = 4), mepolizumab (*n* = 2) and benralizumab (*n* = 1). These treatments seemed more common in subjects with a high (57.1%, *n* = 4) or moderate (28.6%, *n* = 2) mucus score compared to subjects without airway mucus plugging (14.3%, *n* = 1). Monoclonal therapeutics were prescribed to some non-smoker asthmatics.

**Table 1:** Demographics and clinical characteristics of the asthma subjects

Characteristic	All (*n* = 51)	Mucus score		
		Zero (*n* = 13)	Low (*n* = 17)	High (*n* = 21)
**Age, years**	54.9 ± 12.3	50.7 ± 13.7	51.3 ± 11.7	59.9 ± 10.6
**Female sex, %**	23 (45.1)	4 (30.8)	9 (52.9)	10 (47.6)
**BMI, kg/m** ^**2 a**^	28.1 ± 5.0	27.7 ± 4.0	30.0 ± 5.4	26.7 ± 4.8
**Atopic** ^**b**^	32 (68)	8 (61.5)	11 (64.7)	15 (71.4)
**Mucus score**	3 [0.25; 6.25]	0	1.5 [0.5; 3]	7 [5; 9]
**Smoking status** ^**b**^				
Non smoker	28 (59.6)	8 (72.7)	7 (43.7)	13 (65)
Former Smoker	10 (21.3)	2 (18.2)	4 (25)	4 (20)
Current smoker	9 (19.1)	1 (9.1)	5 (31.3)	3 (15)
**Spirometry**				
FEV_1_ pre-BD, %_pred_^a^	64.5 [50.2; 87.5]	67 [55; 89.2]	79.5 [63; 89.5]	56 [40.5; 67.7]
FVC pre-BD,%_pred_^a^	81.7 [66.7; 95.2]	85.5 [71.5; 89.5]	96 [71.5; 104.5]	72.5 [65.7; 93.5]
FEV_1_ /FVC %^a^	63.5 [57.2; 73.7]	70 [61.6; 80.6]	68.4 [60.9; 75.1]	57.1 [46.2; 67.3]
**FeNO, ppb** ^**c**^	19 [9; 27]	12.5 [10.5; 22.1]	14 [6.7; 19.2]	22 [19; 39]
**Sputum eosinophils, %** ^**d**^	1.6 [0.2; 4.7]	0.5 [0; 1.71]	1.2 [0.6; 3.6]	4.7 [0.9; 14]
**Sputum neutrophils, %** ^**d**^	61.5 [50.5; 77.6]	51.2 [31.5; 60.1]	52.1 [50.7; 66.1]	77.6 [59.2; 87.8]
**Gal-10, ng/ml** ^**e**^	16.5 [0, 45.5]	7.5 [1.1; 18.0]	17.1 [3.1; 27.3]	19 [0; 65.6]

Data are presented as mean ± SD, n (%), median [IQR]; BMI= body mass index; FeNO: Fractional Exhaled Nitric Oxide; ICS= inhaled corticosteroids. Missing data: ^a^ n= 3; ^b^ n=4; ^c^ n=33; ^d^ n=26; ^e^ n=23

### Mucus plugs are homogeneously distributed among lung segments and are associated with increased airflow obstruction

**Fig. 2:**
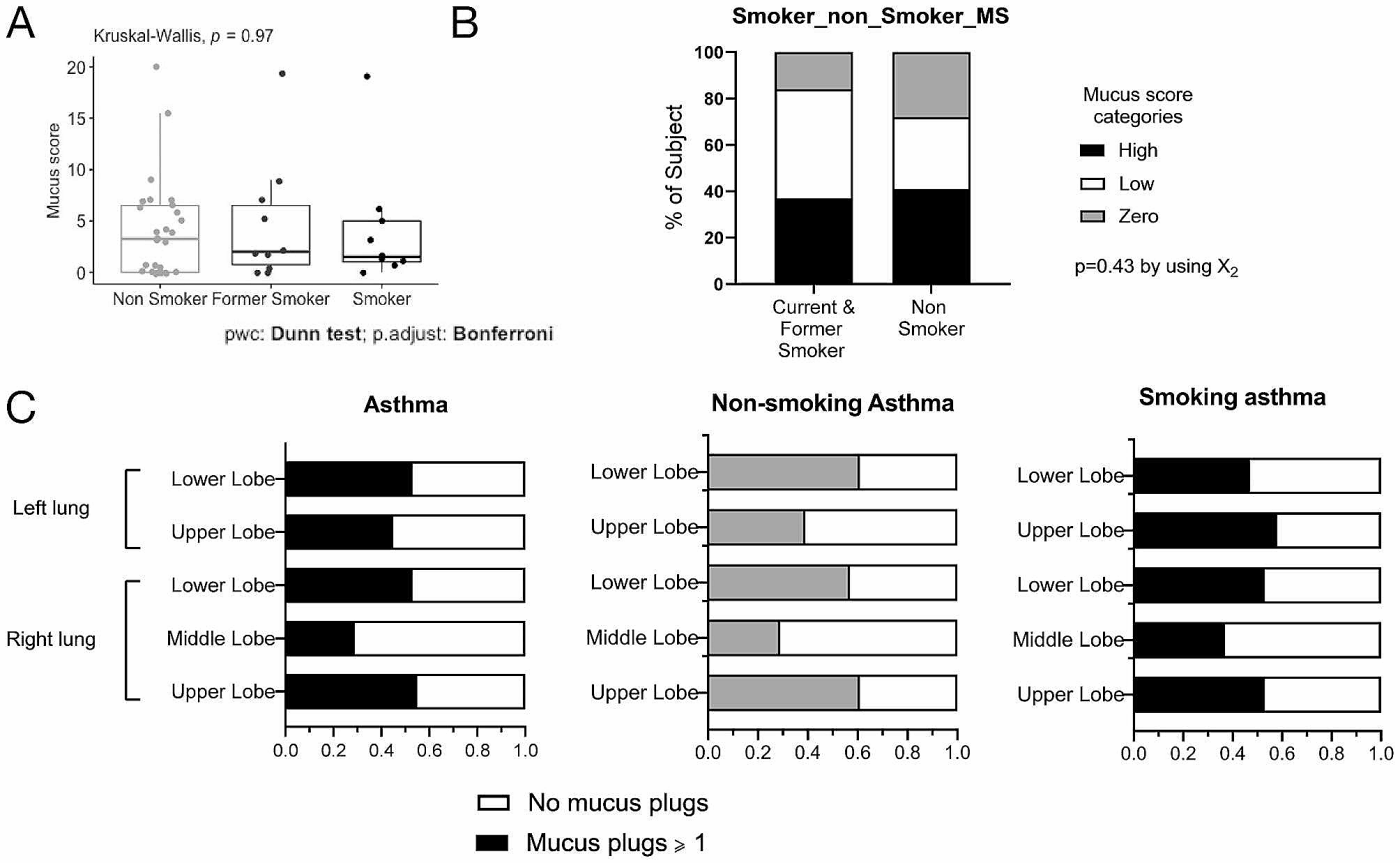
Distribution and localization of mucus plugs in the airways of asthmatic patients according to their smoking status. Panel (**A**) Airway mucus plugs are present in the airways of asthmatic subjects regardless of their smoking status (*n* = 47). Panel (**B**) Distribution of asthmatic patients in the different mucus score classes according to their smoking status (non-smoking or former/active asthmatics) (*n* = 47). Panel (**C**) The distribution of mucus plugs in the different lung lobes, reflected by the percentage of lobes with at least one mucus plug in the lung segments that contain it is homogeneous and does not vary according to smoking status (*n* = 275 lobes analyzed)

The number of asthmatics with at least one airway obstruction by a mucus plug was 38, for a prevalence of 74%. The median mucus score was 3 [0.25; 6.25] and 0.5 [0.37; 0.5] in asthmatics and in the healthy subjects, respectively (Table 1, Supplemental Table 1). The mucus score was not significantly different based on smoking status (Table 2; Fig. 2A). The prevalence of mucus plugs was very common regardless of smoking status with respectively at least one mucus plug in 71% (*n* = 20), 80% (*n* = 8) and 88.9% (*n* = 8) of asthmatic non-smokers, asthmatic former smokers and asthmatic current smokers. The proportion of non-smokers and smoker/former-smoker asthmatics with a “high” mucus score was 41% and 37%, respectively (*p* = 0.4, Fig. 2B).

**Table 2:** Demographics and clinical characteristics of asthmatics among smoking status

	Non Smokers (*n* = 28)	Smoker (*n* = 9)	Former Smoker (*n* = 10)
**Age**	55.4 ± 12.6	46.5 ± 11.4	62 ± 8.9
**Female sex, %**	10 (35.7)	4 (44.4)	7 (70)
**Height (m)** ^a^	1.69 ± 0.1	1.72 ± 0.07	1.64 ± 0.1
**BMI, kg/m** ^**2** a^	27.9 ± 4.8	27.7 ± 5.2	28.4 ± 5.8
**Atopic**	22 (78.6)	5 (55.4)	5 (50)
**Mucus score**	3.25 [0; 6.5]	1.5[1; 5]	2 [0.75; 6.5]
**Spirometry**			
FEV_1_(%pred) ^a^	64 [42; 87]	67 [45; 85]	63 [59.7; 87.5]
FVC (% pred) ^a^	79[61; 93]	88[72; 103]	82.5 [72.2; 101]
FEV_1_/FVC (%)^a^	66.2 [57; 75]	60 [47; 73]	66.5 [61.7; 70.9]
**FeNO, ppb** ^b^	21 [12; 33]	7 [6–9]	16[13.5; 25]
**Sputum eosinophils, %** ^c^	2.5 [0.2; 10.4]	0.7 [0.1; 1.5]	1.5 [1.0; 2.9]
**Sputum neutrophils %** ^c^	53.7 [44.2; 86.6]	63 [53.5; 74.4]	66.2 [50.5; 68.8]
**Gal-10, ng/ml** ^**d**^	16.9[3.4; 64.1]	0[0; 22.2]	23.9[22.2; 25.6]

Data are presented as mean ± SD, n (%), median [IQR]; BMI= body mass index; FeNO: Fractional Exhaled Nitric Oxide; ICS= inhaled corticosteroids. Missing data ^a^ n=2 ^b^ n=14 ^c^ n=22 ^d^ n=19

The overall CT scan analysis included 1100 lung segments, 214 (19.4%) of which demonstrated at least one mucus plug. The distribution was homogeneous in all segments regardless of smoking status (Fig. 2C*).*

**Fig. 3:**
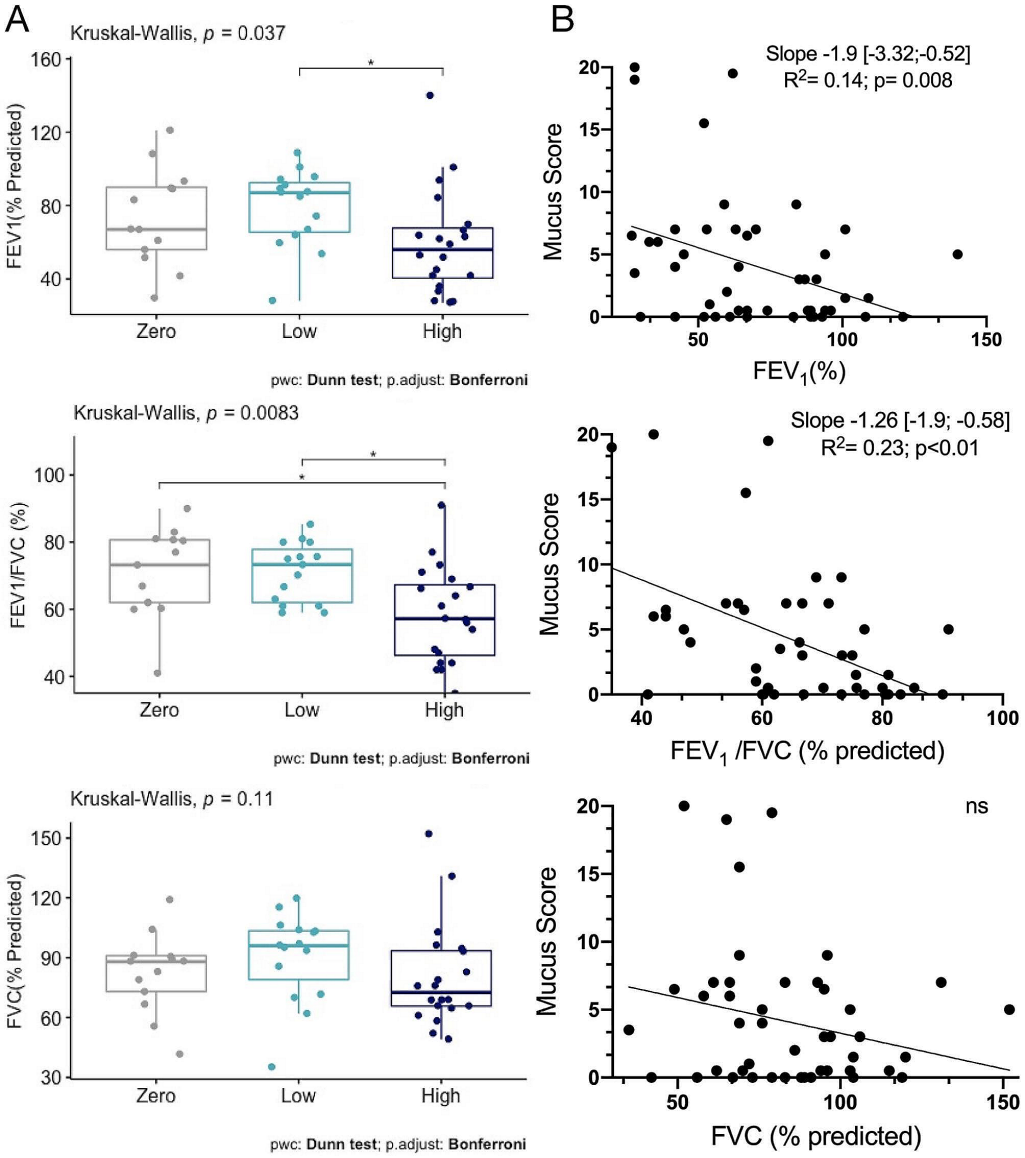
Association between mucus score and airway obstruction in asthmatic patients. Panel (**A**) Comparison of spirometric values according to mucus score class. Panel (**B**) Correlation between mucus score and respiratory function assessed by spirometry in asthmatics

In this cohort, in agreement with previously published studies [4], asthmatics with a high mucus score had more severe airway obstruction than other mucus score classes (Fig. 3A). Moreover, inverse correlations between bronchial obstruction, evaluated by the FEV_1_/FVC ratio and FEV_1_ (% of the predicted) were confirmed with a Spearman’s ρ coefficient of − 0.32 (*p* = 0.02) and − 0.43 respectively (*p* = 0.003). No correlation between FVC and mucus score was found (data not shown). In univariate analysis, FEV_1_ and FEV_1_/FVC respectively were correlated with the mucus score with a slope and a respective R^2^ of -1.9 [-3.32; -0.52]; R^2^ = 0.14 (*p* < 0.01) and − 1.26 [-1.9; 0.59]; R^2^ = 0.23 (*p* < 0.01) (Fig. 3B). In the subgroups of non-smoking asthmatic or former/active smoker asthmatics, these correlations were not found (data not shown*).*

### Airway mucus plugging is associated with type 2 inflammation in asthmatic non-smokers and possibly neutrophilic inflammation in active smokers or former smoking asthmatics

**Fig. 4:**
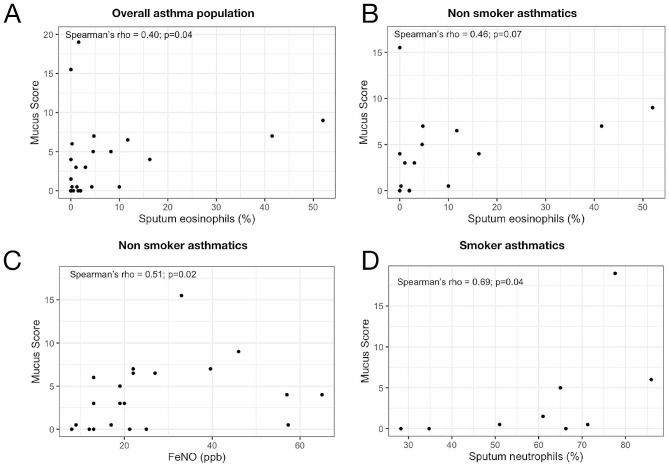
Correlation between the asthmatics airways inflammation and the mucus score according to smoking status. Correlation between the percentage of eosinophils in sputum and mucus score in overall asthma population (*n* = 25). Panel (**A**) Correlation between the percentage of eosinophils in sputum and mucus score in non-smoking asthmatics (*n* = 14). Panel (**B**) Correlation between FeNO and mucus score in the non-smoking asthmatic population (*n* = 21). Panel (**C**) Correlation between sputum neutrophils percentage in active smokers or former asthmatic smokers with the mucus score (*n* = 9). Correlations were performed using Spearman’s parametric correlation test

In the overall population of asthmatic subjects, the mucus score was positively and significantly correlated with the percentage of eosinophils in the sputum (Spearman’s ρ = 0.4; *p* = 0.04, Fig. 4.A). In non-smoking asthmatics, this correlation tended towards confirmation of this relationship (Spearman’s ρ = 0.46, *p* = 0.07, Fig. 4.B) but not in active or former smoking asthmatics (Spearman’s ρ = 0.26, *p* = 0.49). No association between mucus score and blood eosinophilia or serum IgE level was demonstrated (data not shown). FeNO was positively and significantly associated with the mucus score in non-smoking asthmatic subjects (Spearman’s ρ = 0.51; *p* = 0.02, Fig. 4.B). This correlation was lost in the total asthmatic cohort (*p* = 0.17) and in former/current asthmatic smokers (*p* = 0.17). In the group of asthmatic subjects who were former or active smokers, the percentage of neutrophils in sputum was positively and significantly associated with mucus scores (Spearman’s ρ = 0.69, *p* = 0.04; Fig. 4.D). This association was not demonstrated in the overall asthmatic cohort or in non-smoking subjects (data not shown).

### Gal-10 10 in the sputum of asthmatic subjects is a marker of type 2 inflammation but not of the occlusion of the airways by mucus plugs

Confirmation of an association between sputum eosinophilia, T2-type inflammation and the presence of mucus plugs in the airways of asthmatic subjects prompted us to investigate the involvement of Charcot Leyden crystals in plug formation, as suggested by other authors [8]. A surrogate marker, Gal-10, which is the protein component of these crystals, was measured in the available sputum supernatants (40 asthmatic subjects and 3 healthy subjects). In asthmatics, the median Gal-10 concentration was 27.3 ng/ml [2.3; 84.4].

**Fig. 5:**
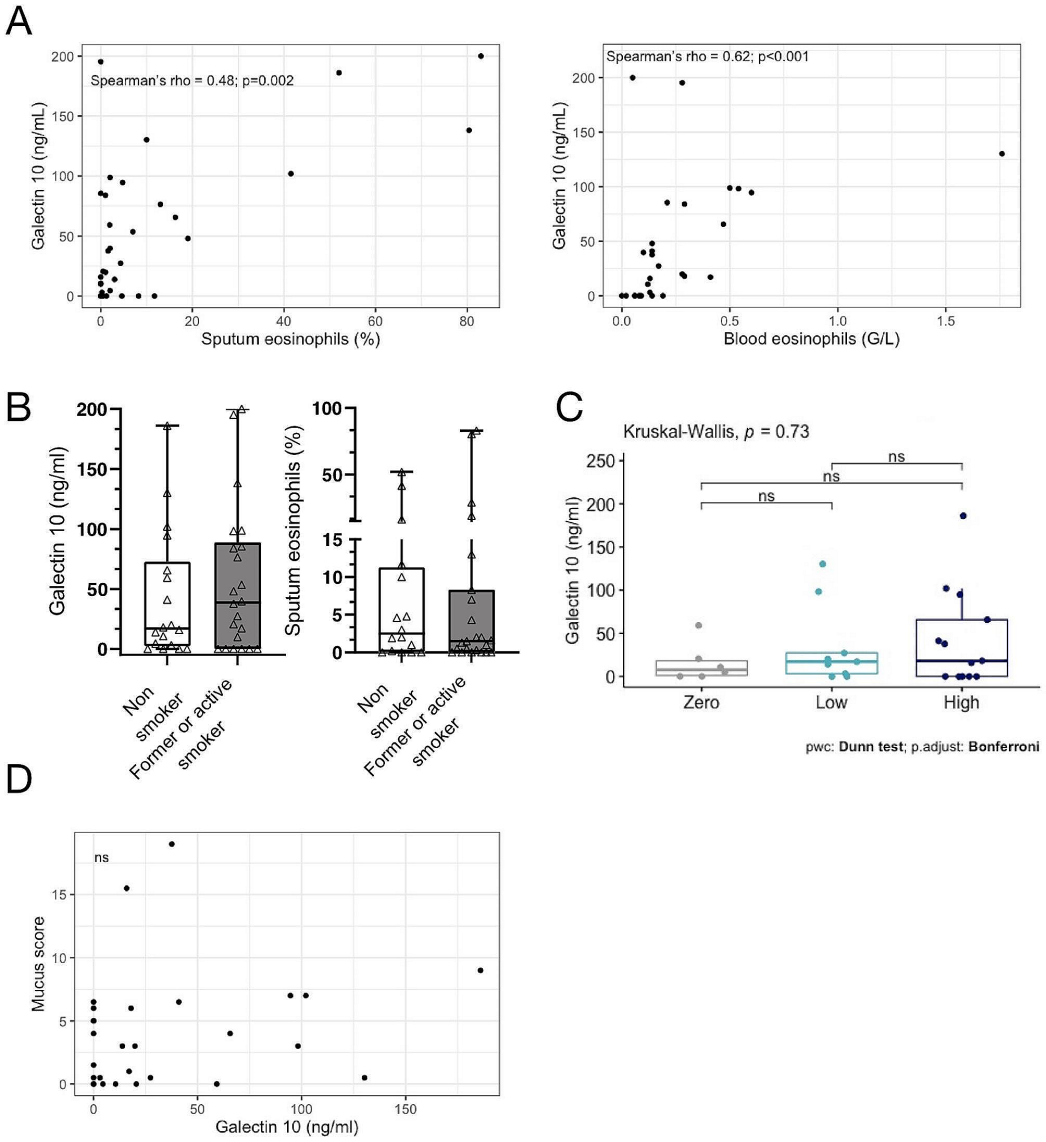
Association of sputum Gal-10 concentration and airways eosinophilic inflammation or airway mucus plugging. Association of sputum concentration of Gal-10 and the percentage of eosinophils in the sputum and in the blood in asthmatics (respectively *n* = 30 and *n* = 26). Panel A. Comparison of the concentration of Gal-10 and the percentage of eosinophils in the sputum of subjects who are ex/active smokers (*n* = 16 and *n* = 16) and asthmatic non-smokers (*n* = 23 and *n* = 22). Panel (**A**) Comparison of Gal-10 concentration in sputum of asthmatic subjects according to mucus score groups (*n* = 31). Panel (**B**) Association of Gal-10 concentration and mucus score in asthmatic subjects (*n* = 26). Correlations were performed using Spearman’s parametric correlation test

The concentration of Gal − 10 in the sputum of asthmatics was correlated with the percentage of eosinophils in the sputum (Spearman’s ρ = 0.48, *p* = 0.002, Fig. 5.A) and with blood eosinophilia (Spearman’s ρ = 0.85, *p* < 0.001 Fig. 5.B). These associations were even stronger in non-smoking asthmatics with a Spearman’s ρ of 0.58 (*p* = 0.01) and 0.95 (*p* < 0.001), respectively. No other association between Gal-10 concentration and type 2 parameters (IgE and FeNO) was demonstrated (data not shown). In current or former asthmatic smokers, sputum Gal-10 concentrations tended to be higher with no apparent change in eosinophil concentration between these two groups (Fig. 5.C). The percentage of neutrophils in sputum was negatively correlated with Gal-10 concentration (Spearman’s ρ = -0.36, *p* = 0.02). We explored the link between the concentration of Gal-10 in sputum and the presence of mucus plugs in the bronchi but no significant difference in concentration was demonstrated among the different categories of mucus scores (Fig. 5.D). No correlation was found with mucus score and the quantity of Gal-10 in sputum (Spearman’s ρ = 0.11, *p* = 0.53, Fig. 5.E) or other lung function parameters (data not shown). These data suggest that Gal-10 is a marker of eosinophilic inflammation in asthmatics but may not be associated with the formation of mucus plugs. Given an association between sputum eosinophilia and mucus plug formation, this finding suggests that the Gal-10 present in the airways may originate also from other cell types [18, 19].

### Comparison of airway architecture in asthmatics with a high mucus score versus asthmatics with a low or no mucus score

To explore the association between airway architecture and airway mucus plugging, wall thickness and lumen area were compared between CT scans of asthmatics with a high mucus score and scans of asthmatics with low or zero mucus score. CT scans of 40 asthmatics were included in the analysis. This analysis could not be carried out on the remaining scans for technical reasons. The different dimensions of the airways and subject characteristics are reported in table S2 and S4. The comparisons of these characteristics were adjusted for the height, sex and age of the subjects and analyzed at different levels of the bronchial tree, from trachea to segmental level. After adjusting for age, sex, height of the subjects, the lumens of the main bronchi and the lobar bronchi were significantly inversely correlated with the high mucus score with β coefficient at **-**1.18 (-1.89, -0.47; *p* = 0.001) and − 0.77 (-1.39, -0.15; *p* = 0.015), respectively (Table 3). The wall thickness was also significantly and inversely correlated with a high mucus score in asthmatics [ρ = -0.16 (-0.29, -0.03; *p* = 0.015; Table 3)] in the mainstem bronchi. No association was found between WA% and a high mucus score (Table 3).

**Table 3:** Correlation between airway dimensions according to the presence of a high mucus score after adjusting for age, height and sex (*n* = 40)

	Lumen Diameter	Wall Thickness	%WA
**Trachea**			
mucus score	-0.71 (-1.99, 0.58) *P* = 0.283	0.01 (-0.15, 0.17) *P* = 0.917	0.87 (-1.33, 3.06) *P* = 0.440
**Main bronchi**			
mucus score	**-1.18 (-1.89, -0.47)** *P* = 0.001	**-0.16 (-0.29, -0.03)** *P* = 0.015	0.31 (-1.26, 1.88) *P* = 0.699
**Lobar**			
mucus score	**-0.77 (-1.39, -0.15)** *P* = 0.015	-0.14 (-0.36, -0.08) *P* = 0.215	0.68 (-3.16, 4.54) *P* = 0.726
**Segmental**			
mucus score	-0.14 (-0.47, 0.20) *P* = 0.435	-0.09 (-0.19, 0.01) *P* = 0.089	0.07 (-2.56, 2.70) *P* = 0.957

After replacing the high mucus score variable by the total mucus score, the negative correlation between mucus score and the lumen diameter and the wall thickness of the mainstem bronchi remained significant (Table S3). In our asthmatic population, after controlling for confounding factors (the size, height, sex and age), the lumens of the main bronchi and the lobar bronchi were inversely associated with the mucus score.

## Discussion

In our cohort that was mainly composed of severe asthmatics (*n* = 44; 86%), 74% of subjects had at least one airway mucus plug. Previous correlations between the mucus score and airflow obstruction, and with the percentage of sputum eosinophils were confirmed in our overall asthmatic cohort. A principal contribution of our study is that mucus plugs are present in the airways regardless of the smoking status of the asthmatic subjects. However, the correlation between the mucus score and the airway inflammation (percentage of eosinophils or neutrophils in sputum) appears to differ depending on smoking status. Consistent with previously published studies [4, 20, 21], in non-smoking asthmatics, we found that the percentage of sputum eosinophils was associated with airway mucus plugging. This correlation was not found in asthmatic smokers/former smokers. In this group, the mucus score was correlated with the percentage of neutrophils in the sputum. The second principal finding of our study is the association between airway architectural modifications in subjects with a high mucus score compared to those with a low or zero mucus score. Asthmatics with many airway mucus plugs had narrower mainstem bronchi and lobar bronchi compared to subjects with a low or zero score. Furthermore, in these sub-populations, the wall thickness of the main bronchus was reduced compared to subjects with a moderate or zero mucus score.

This work offers an external validation of the results derived from the SARP cohort [4, 5]. The prevalence of mucus plugs was 71%, 80% and 88.9% of asthmatic non-smokers, asthmatic former smokers and asthmatic current smokers compared to reported values of 58-100% in asthma [4, 20, 21], 57% in COPD [2] and 65% for ACO [21]. The prevalence of mucus plugs in our study was higher than in previously published studies [4, 5]. This discrepancy could be due to the characteristics of the population included in the study. The prevalence of severe asthma in our population is 86% vs. 65.8% in the study by Dunican et al. [4]. All of these data suggest that mucus plugs are a marker of asthma severity. In this cohort, the distribution of these plugs throughout the airways was homogeneous and was not affected by smoking status. A predominance of airway mucus plugging in the lower lobes has been demonstrated in subjects with asthma [5] and COPD [22]. This finding suggests an importance for gravity-dependent airway narrowing. However, this predominance was not systematically found in other cohorts [2, 4].

Regarding the association between airway mucus plugging and airway obstruction, we found a weak but significant correlation between mucus score and FEV_1_ and FEV_1_/FVC ratio. Several factors could have weakened the strength of these correlations despite using the same methodology. Firstly, exposure to cigarette smoke may have caused airflow obstruction by mechanisms other than airway mucus plugging, such as small airway inflammation or emphysema [23]. Secondly, the severity of asthma and the use of high-dose inhaled corticosteroids may have influenced these correlations. These discrepancies suggest that airway obstruction in severe asthmatics has determining factors other than mucus plugs. Furthermore, the lung subtended by these plugs is relatively small making it improbable that they are directly responsible for the overall degree of obstruction assessed by spirometry. The lack of an association with FVC, and therefore indirectly air trapping, supports the idea that mucus plugs are not causing a substantial lung volume inaccessible to inspired air. However, a relationship between mucus plugs on CT scans and ventilatory defects on MRI imaging has been reported [7]. It is probable that they may contribute to some of the observed ventilation to perfusion mismatching. As suggested for COPD [24], airway mucus plugs in asthma may be considered as a phenotypic biomarker for disease severity.

Thus, it is important to emphasize that the parameters associated with the presence of these markers are multiple and may vary depending on the clinical context. In non-smoking asthmatics, the association between mucus plugs and eosinophilic inflammation is well established. In contrast, in asthmatic smokers or former smokers, an association with neutrophilic inflammation is suggested. 53% of subjects without eosinophilic inflammation in the sputum contemporary to the CT scan had at least one mucus plug. These results are consistent with a correlation between the mucus score and percentage of neutrophils in sputum in COPD subjects [2] and an inverse correlation between the same score and the percentage of eosinophils in sputum in asthma/COPD overlap subjects [21]. This difference suggests that the mechanisms underlying the presence of mucus plugs in the airways may be different according to the smoking status. However, it is possible that the subjects with mucus plugs had prior eosinophilic inflammation leading to the plug formation since sputum characteristics are not always stable over time [25].

The concentration of soluble Gal-10 in sputum did not correlate with the mucus score regardless the smoking status. Charcot-Leyden crystals have been proposed to be integral to the structure of the plug and to contribute to its formation by the physicochemical properties of Gal-10, an eosinophil granule protein [8]. Crystalline Gal-10 has an adjuvant function, promoting T2 immunity and antibody dissolution of the crystalline form prevents this function [8]. Thus, by promoting T2 inflammation a relationship with mucus plug formation would be expected but was not confirmed in our work. Before refuting the hypothesis, it should be noted that in vitro, Gal-10 in its soluble state does not induce an immune response [8]. This characteristic is only found in the crystalline form. For technical reasons, we were not able to search for Charcot-Leyden crystals in sputum. However, the sputum concentration of Gal-10 could be used as a surrogate marker for evaluating type 2 bronchial inflammation as suggested by other studies [26, 27]. Whether the dissolution of the mucus plug in human in vivo will result from the depolymerization of Gal-10 as has been proposed [8] will require further study.

Beyond bronchial inflammation, Tran et al. have found that the airway wall thickness was increased in segments with distal mucus plugs [3]. With a similar methodology, after adjusting for height, sex, and age, asthmatics with a high mucus score had reduced main bronchial and lobar bronchial calibers. These findings suggest that airway architecture may promote airway mucus plugging or that this association may be a consequence of a local process leading to airway mucus plugging and modification of airway characteristics. The findings also suggest that the severity of airflow limitation is related to airway narrowing detectable by CT scanning. The finding of thinner airways is not explained. However, if these subjects experienced a greater degree of hyperinflation any accompanying lengthening of the airways would be expected to cause thinning.

An additional factor that may alter the probability of mucus plugging is the efficacy of mucociliary clearance, which is impaired in asthma [28] and T2 inflammation is associated with greater impairment than non-T2 inflammation [29]. Our association between mucus plugging and neutrophilic inflammation or modification of the airways architecture could be related to a recent identification of a new phenotype of severe asthma defined by a modification of ciliary gene expression with an airways neutrophilic inflammation and increased mucus production [30].

This study has several limitations. The first is the presence of missing or unmatched data due to the different design of the two studies (Difficult-to-treat asthma and ACO study). The second limitation is the length of patient recruitment spanning between 2012 and the present. Nevertheless, all the clinical and functional correlations were always carried out within an interval of 1 month between the chest CT scan and the respiratory function. The weak associations between mucus plug formation and lung function may have been strengthened by tests that are more sensitive to small airway dysfunction, such as may be acquired by oscillometry. Third, the Gal-10 sputum analysis concurrent with chest CT assessment was only available for a limited number of patients. The sputum treatment may have dissolved the Gal-10 crystals and participated in the cytolysis of the eosinophils. If so, the phenomenon may have increased the quantity of immunoreactive Gal-10 in some samples. In addition, the impact of the use of SSI and DTT on the Gal10 dosage has not been specifically studied and could have modified the quantity of protein found in sputum.

In conclusion, mucus plugs in the airways are a phenotypic marker of asthma. The presence of mucus plugs is most frequently associated with T2 inflammation, but we have not been able to demonstrate an association between Gal-10 concentration in sputum and the number of mucus plugs. However, in a minority of patients, mucus plugs could be associated with other determinants than T2 inflammation such as bronchial architecture or neutrophilic inflammation.

### Electronic supplementary material

Below is the link to the electronic supplementary material.

Supplementary Material 1

## Data Availability

Data and materials relating to this study will be made available upon request.

## Abbreviations

BMI: Body Mass Index
COPD: Chronic obstructive pulmonary disease
FeNO: Fractional exhaled nitric oxide
FEV1: Forced Expiratory Volume in one second
FVC: Forced vital capacity
Gal-10: Galectine-10
WA: Wall area
