# Supplementary material for: Mucus plugs in the airways of asthmatic subjects and smoking status
Source: Respir Res. 2024 Jan 23;25:52. doi: 10.1186/s12931-024-02665-w (PMC10807136; doi:10.1186/s12931-024-02665-w)
Supplement: Supplementary file 1 — Supplementary Material 1 [file 12931_2024_2665_MOESM1_ESM.docx]

Mucus plugs in the airways of asthmatic subjects and smoking status.

**Table S1:** Healthy subject demographics

| **Characteristic** | **Healthy**  **(n=5)** |
| --- | --- |
|  |  |
| **Age, y** | 42,1 ± 12,3 |
| **Female sex, %** | 1 (20%) |
| **BMI, kg/m^2^** | 29 ± 8,6 |
| **Atopic** | 3 (60) |
| **Mucus score** | 0.5 [0; 0.5] |
| **Non smoker** | 4 (100%) |
| **FEV_1_ pre-BD, %_pred_** | 99,4 ± 12 |
| **FVC pre-BD,%_pred_** | 100 ± 11,9 |
| **FEV_1_ /FVC %** | 81 ± 5 |
| **FeNO, ppb ^a^** | 13 [12-16] |
| **Sputum eosinophils ^c^, % ^a,b^** | 0 [0-0.12] |
| **Sputum neutrophils ^a,b^, % ^a^** | 37.2 [18.6;62] |
| **G-10, ng/ml ^a,b^** | 66.3 [610.5; 134,6] |

^a^ median [IQR]; ^b^ n=3

**Table S2**: Airways dimension (in millimeter) among asthmatics

|  | **Lumen Diameter** | **Wall Thickness** | **%WA** |
| --- | --- | --- | --- |
| Trachea | 16.5 (2.2) | 2.6 (0.3) | 40.3 (3.2) |
| Large bronchi (RMB, Bronch Int, LMB) | 10.8 (1.5) | 2.2 (0.3) | 46.4 (2.6) |
| Lobar (RUL, RLL, LLB6) | 7.4 (1.0) | 2.0 (0.3) | 53.4 (4.9) |
| Segmental (RB1, RB4, RB10, LB1, LB10) | 4.1 (0.5) | 1.6 (0.2) | 64.3 (3.9) |

n = 40 asthmatics, result as mean (SD) in millimeter

**Table S3:** Correlation between airway dimensions according to the mucus score after adjusting for age, height and sex

|  | **Lumen Diameter** | **Wall Thickness** | **%WA** |
| --- | --- | --- | --- |
| **Trachea** | -0.05 (-0.18, 0.07)  P=0.424 | -0.01 (-0.02, 0.01)  P=0.474 | 0.02 (-0.19, 0.24)  P=0.840 |
| **Main stems** | **-0.10 (-0.17, -0.03)**  **P=0.005** | **-0.02 (-0.03, -0.01)**  **P=0.001** | -0.01 (-0.16, 0.14)  P=0.862 |
| **Lobar** | -0.04 (-0.10, 0.02)  P=0.174 | -0.01 (-0.03, 0.01)  P=0.184 | -0.06 (-0.40, 0.29)  P=0.755 |
| **Segmental** | -0.001 (-0.03, 0.03)  P=0.965 | 0.002 (-0.01, 0.01)  P=0.731 | 0.02 (-0.02, 0.26)  P=0.861 |

**Table S4:** Demographics and clinical characteristics of asthmatics included in the airway architectural analysis

|  | **Asthmatics**  **(n=40)** |
| --- | --- |
| **Age** | 55.3 **±**12.2 |
| **Female sex, %** | 47.6 **±** (20) |
| **Height (m)** ^a^ | 1.69 **±** 0.3 |
| **BMI, kg/m^2^** ^a^ | 28.3 **±** 4.9 |
| **Atopic^e^** | 73.4 (29) |
| **Smoking status** |  |
| **Non smoker** | 22 |
| **Active** | 8 |
| **Former** | 9 |
| **Mucus score** | 2.5 [0.5; 6.3] |
| FEV_1_ (%pred) ^a^ | 67.2 **±** 25.9 |
| FVC (% pred) ^a^ | 81.9 **±** 23.4 |
| FEV_1_/FVC (%)^a^ | 65.3 ± 18.5 |
| **FeNO, ppb** ^b^ | 19 [9; 33] |
| **Sputum eosinophils, %** ^c^ | 1.5 [0; 4.6] |
| **Sputum neutrophils %** ^c^ | 61.2 [50.6; 83.3] |
| **Galectin 10, ng/ml^d^** | 35.6 [0; 52.2] |

Data are presented as mean ± SD

Missing data ^a^ n= 2 ^b^ n=13 ^c^ n=7 ^d^ n=9; **^e^** n=3; Spiro missing: 2 Gal missing 3

**Glossary**

|  |  |
| --- | --- |
| RMB | right main bronchus |
| LMB | left main bronchus |
| RUL | right upper lobe |
| Bronch Int | bronchus Intermedius |
| RLL | right lower lobe |
| LLB6 | lower bronchus number 6 segment |
| %WA | Percentage of wall area |
